# A innovative prognostic symbol based on neutrophil extracellular traps (NETs)-related lncRNA signature in non-small-cell lung cancer

**DOI:** 10.18632/aging.203289

**Published:** 2021-07-13

**Authors:** Chen Fang, Fen Liu, Yong Wang, Shangkun Yuan, Renfang Chen, Xiaotong Qiu, Xiaoying Qian, Xinwei Zhang, Zhehao Xiao, Qian Wang, Biqi Fu, Yong Li

**Affiliations:** 1 Department of Medical Oncology, The First Affiliated Hospital of Nanchang University, Nanchang 330000, China; 2 Critical Care Medicine, The First Affiliated Hospital of Nanchang University, Nanchang 330000, China; 3 Department of Rheumatology, The First Affiliated Hospital of Nanchang University, Nanchang 330000, China

**Keywords:** non-small-cell lung cancer, neutrophil extracellular traps, lncRNA, prognosis

## Abstract

Neutrophil extracellular traps (NETs) are closely related to cancer progression. NETs-related lncRNAs play crucial roles in non-small-cell lung cancer (NSCLC) but there have been no systematic studies regarding NETs-related long noncoding RNA (lncRNA) signatures to forecast the prognosis of NSCLC patients. It’s essential to build commensurate NETs-related lncRNA signatures. The expression profiles of prognostic mRNAs and lncRNAs and relevant clinical data of NSCLC patients were downloaded from The Cancer Genome Atlas (TCGA) database. The NETs-related genes came from the results of our transcriptome RNA microarray data. The co-expression network of lncRNAs and NETs-related genes was structured to confirm NETs-related lncRNAs. The 19 lncRNAs correlated with overall survival (OS) were selected by exploiting univariate Cox regression (*P* < 0.05). Lasso regression and multivariate Cox regression (*P* < 0.05) were utilized to develop a 12-NETs-related lncRNA signature. We established a risk score based on the signature, which suggested that patients in the high-risk group displayed significantly shorter OS than patients in the low-risk group (*P* < 0.0001, *P* = 0.0023 respectively in the two cohorts). The risk score worked as an independent predictive factor for OS in both univariate and multivariate Cox regression analyses (HR> 1, *P*< 0.001). Additionally, by RT-qPCR, we confirmed that NSCLC cell lines have higher levels of the three adverse prognostic NETs-related lncRNAs than normal lung cells. The expression of lncRNAs significantly increases after NETs stimulation. In short, the 12 NETs-related lncRNAs and their model could play effective roles as molecular markers in predicting survival for NSCLC patients.

## INTRODUCTION

Lung cancer is the leading cause of tumor-related death worldwide and ranks second in incidence among malignancies [1]. Specifically, the proportion of non-small-cell lung cancer (NSCLC) in diagnosed lung cancer cases is approximately 80-85% [2]. Although advances in chemotherapy, radiation therapy, immunotherapy, and targeted therapy have reduced mortality among patients with NSCLC over the years [3], the long-term cancer-specific survival rates have scarcely been increased, especially compared with those of other cancers [4]. Hence, it is essential to probe the molecular mechanisms of NSCLC progression and to explore more precise tumor prognostic markers that accurately predict the survival of patients with NSCLC.

Neutrophils [5], the most affluent endogenous immune effector cells, can desorb modified chromatin structures decorated with given cytoplasmic and granular proteins called neutrophil extracellular traps (NETs) to respond to specific stimuli, mainly via a cell death process termed NETosis [6]. Commonly, NETs trap, neutralize and kill bacteria, fungi, viruses, and parasites and are thought to prevent bacterial and fungal dissemination [7, 8]. Consequently, the generation of NETs and NETosis are deemed evolutionary processes, of which disorder and dysregulation can result in many diseases, such as infection, thrombosis, tissue injury, organ dysfunction, and cancer metastasis [9, 10]. However, the molecular mechanisms of NETs in cancer remain poorly understood. Knowledge about these mechanisms may assist in the development of NETs-focused therapeutic interventions.

Long noncoding RNAs (lncRNAs) [11], recognized as transcripts of more than 200 nucleotides that cannot be translated into proteins, were identified to execute an extensive range of functions in various crucial biological activities, such as cell proliferation and differentiation, genetic regulation of gene expression, action variation of the transcriptome, and microRNA (miRNA) regulation [12]. Importantly, lncRNAs contribute to the development of NSCLC in proliferation, migration, invasion, and chemoresistance [13, 14]. Previous studies have commonly focused on a single or a few lncRNAs for NSCLC [14–16]. Moreover, NETs-related lncRNA expression profiles have not yet been developed to explore novel biomarkers for forecasting the prognosis of NSCLC. Meanwhile, whether NETs encourage the malignant phenotype of NSCLC through some lncRNAs remains largely unknown. Consequently, we aimed to utilize bioinformatics to establish NETS-related lncRNA signatures and to seek new biomarkers to predict the prognosis of patients with NSCLC.

## RESULTS

**Figure 1:**
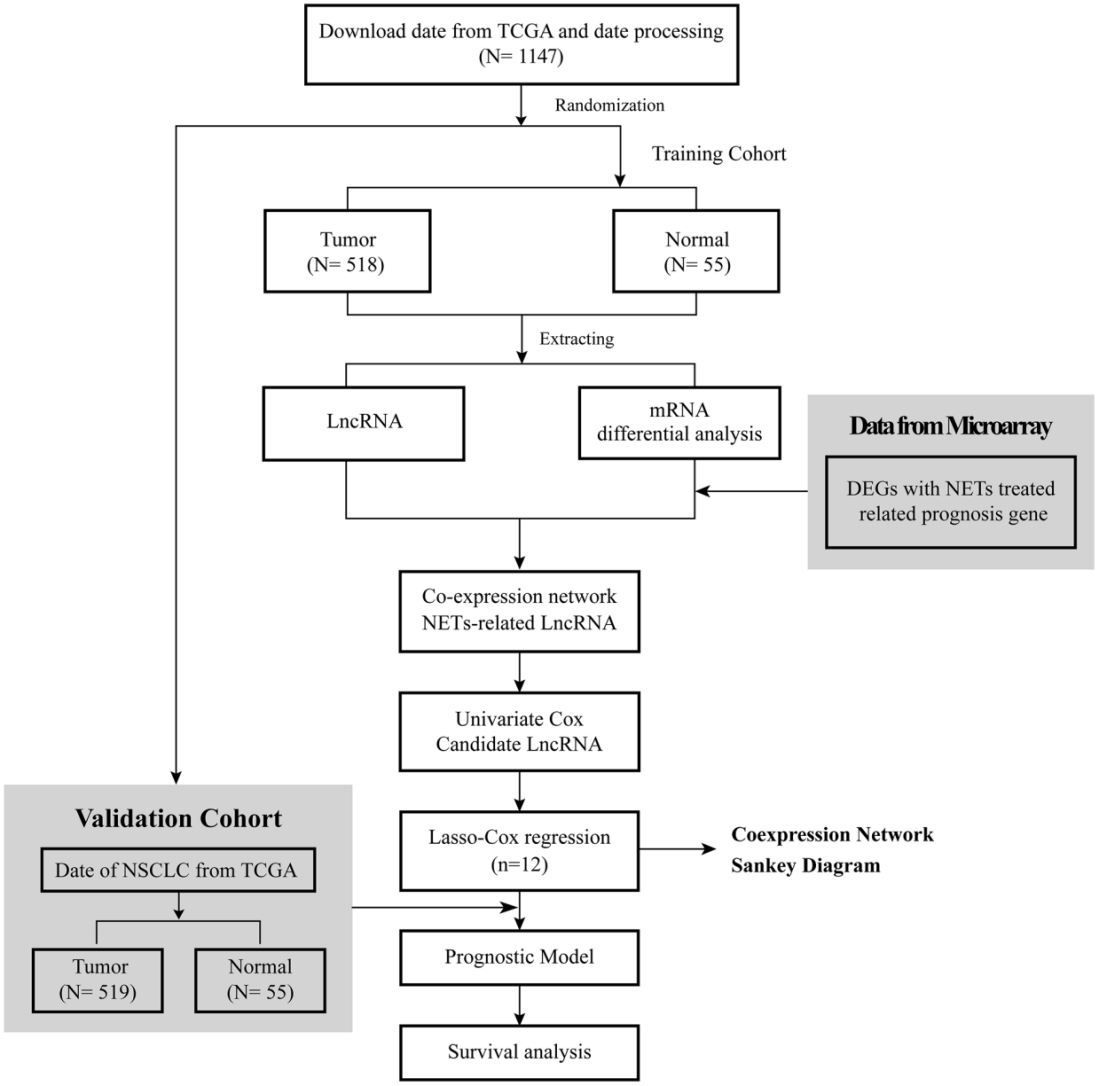
Flow diagram of this research.

The flow diagram of this research is shown in Figure 1. The RNA-seq data of 1037 NSCLC tissue samples, and 108 healthy lung samples were obtained from The Cancer Genome Atlas (TCGA) and were randomly divided into two groups, a training cohort and a validation cohort, were finally enrolled. Meanwhile, the clinical data of 967 NSCLC patients were obtained from TCGA, and their detailed clinical characteristics are shown in Table 1.

**Table 1:** Clinical characteristics of the participants from TCGA in this research.

	**Training group**	**Validation group**
No. of patients	482	485
Age, years (%)		
≤ 65	206 (42.7%)	199 (41%)
> 65	276 (57.3%)	286 (59%)
Gender (%)		
Female	179 (37.1%)	199 (41%)
Male	303 (62.9%)	286 (59%)
Tumor (%)		
T1	140 (29%)	141 (29.1%)
T2	270 (56%)	275 (56.7%)
T3	53 (11%)	47 (9.7%)
T4	19 (4%)	21 (4.3%)
Tx	NA	1 (0.2%)
Lymph Node (%)		
N0	304 (63.1%)	319 (65.8%)
N1	108 (22.4%)	99 (20.4%)
N2	61 (12.7%)	56 (11.5%)
N3	2 (0.4%)	3 (0.6%)
Nx	7 (1.4%)	8 (1.7%)
Metastasis (%)		
M0	358 (72.3%)	354 (73%)
M1	14 (2.9%)	19 (3.9%)
Mx	110 (22.8%)	112 (23.1%)
Stage(%)		
I	257 (53.3%)	266 (54.9%)
II	130 (27%)	121 (24.9%)
III	81 (16.8%)	78 (16.1%)
IV	14 (2.9%)	20 (4.1%)
Survival status		
OS months (median)	27	21.4

No.: number; NA: not available; OS: overall survival.

### Identification of the candidate NETs-related genes

**Figure 2:**
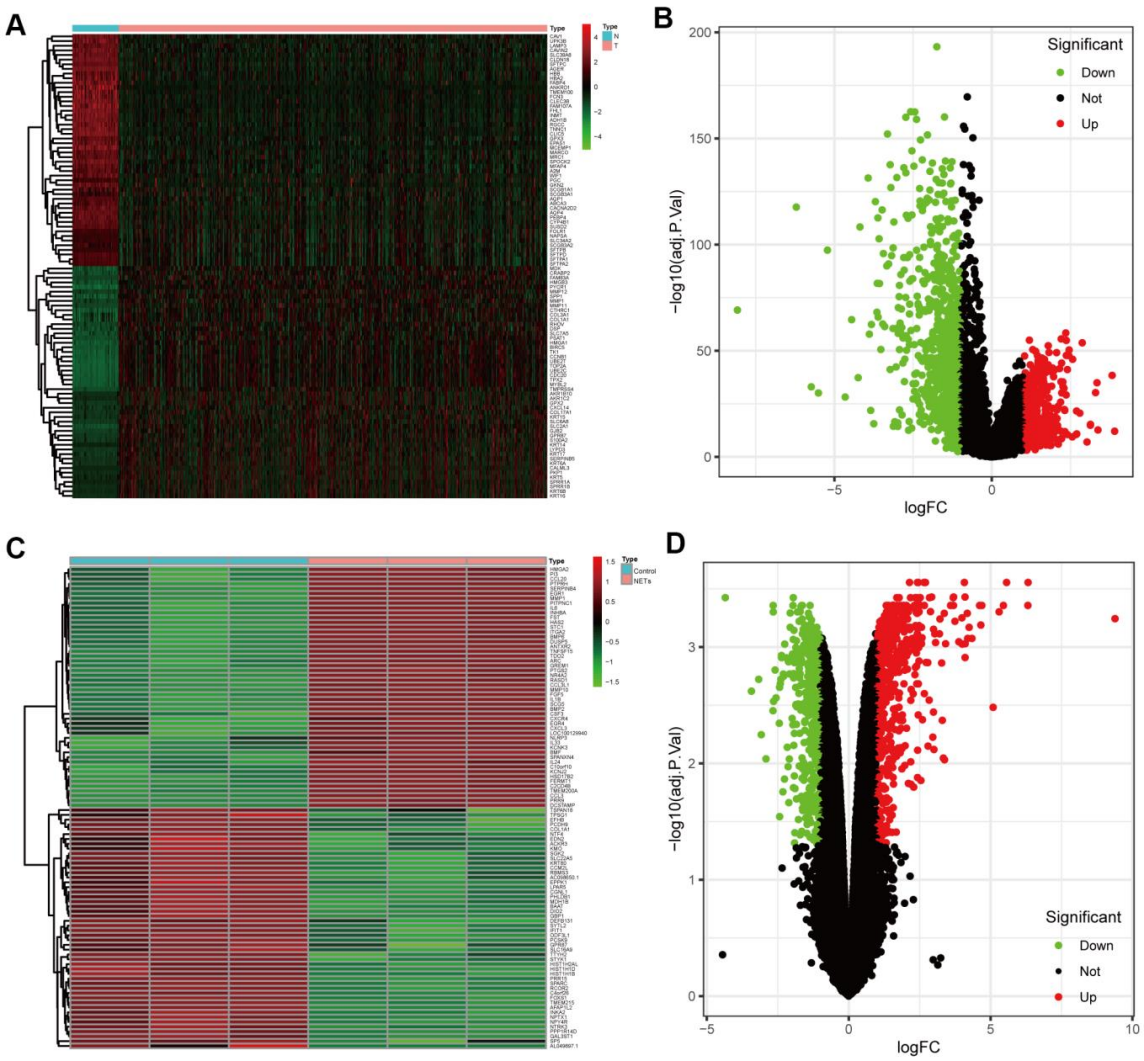
**Identification of the candidate prognostic NSCLC-related differentially expressed genes (DEGs) and NETs-related DEGs.** (**A**, **B**) Heatmap and volcano plot of DEGs between NSCLC patients and healthy people that from TCGA datasets in the training cohort. (**C**, **D**) Heatmap and volcano plot showing DEGs in the transcriptome RNA microarray of A549 cells treated with or without NETs for12 h.

**Figure 3:**
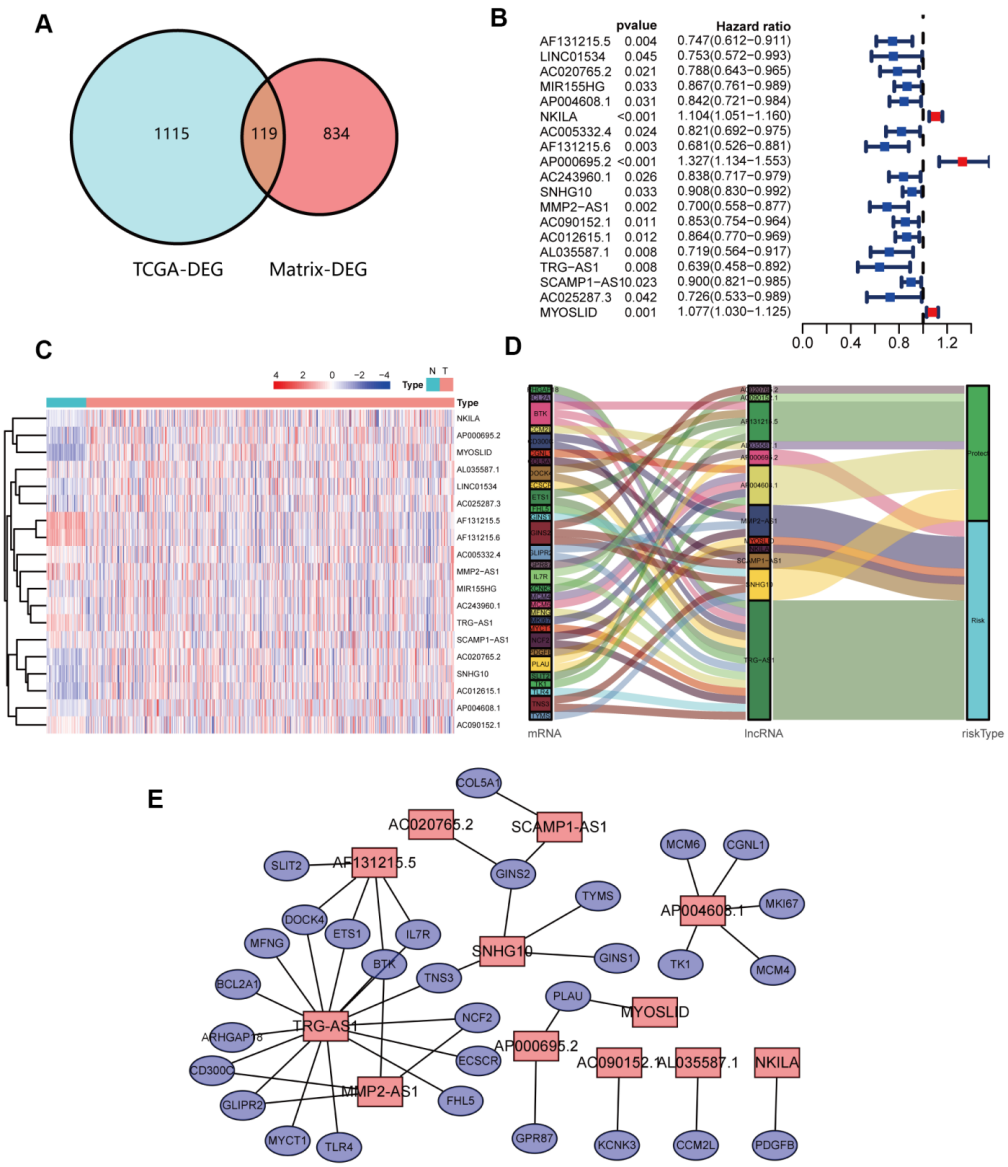
**Identification and analyses of lncRNAs correlated with NETs.** (**A**) Venn diagram identifying 119 overlapping NETs-related genes between TCGA and transcriptome RNA microarrays. (**B**) Forest plots demonstrating the results of the univariate Cox regression analysis between NETs-related lncRNA expression and overall survival (OS). (**C**) Heatmap revealing the NETs-related lncRNAs that were associated with OS by univariate Cox regression analysis in NSCLC patients and healthy people. (**D**) The coexpression network between 12 NETs-related lncRNAs and prognostic DEGs. Red indicates NETs-related lncRNA, and the purple indicates prognostic DEGs. (**E**) Sankey diagram showing the relationships among 12 NETs-related lncRNAs, prognostic DEGs and risk types.

DEGs between NSCLC patients and healthy people from TCGA were analyzed (Figure 2A, 2B). Simultaneously, we obtained the results of DEGs from the transcriptome RNA microarray in the NETs treated and untreated group (Figure 2C, 2D). Then, with | log2 (fold change) | ≥ 1 and FDR < 0.05, we performed a comprehensive analysis on 1115 prognostic NSCLC-related DEGs and 834 NETs-related DEGs, ultimately obtaining 119 genes that were mainly were correlated with NSCLC and NETs (Figure 3A).

### Identification of the prognostic NETs-related lncRNA signature

**Figure 4:**
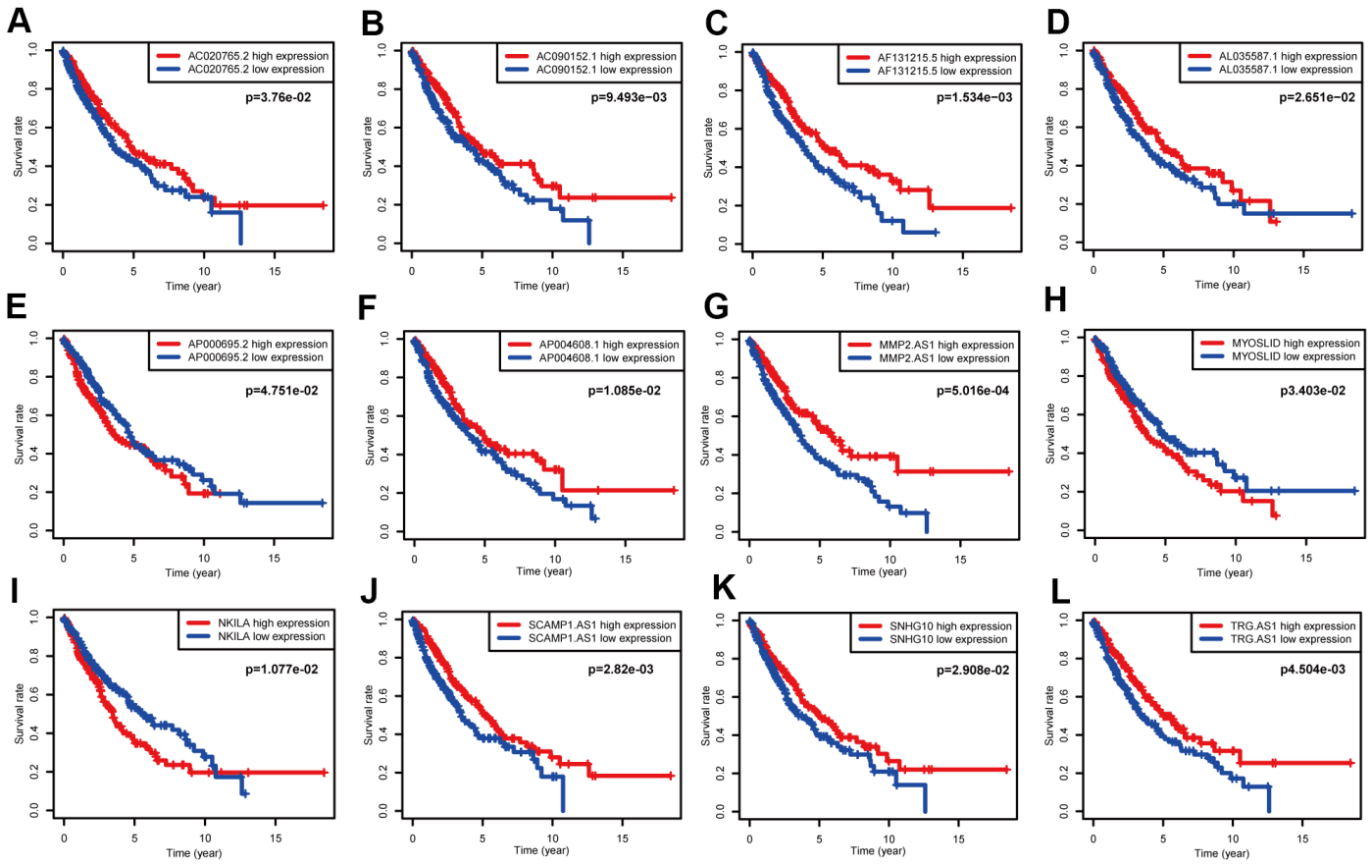
**Survival data analyses of 12 NETs-related lncRNAs in the training cohort.** (**A**–**L**) Kaplan-Meier survival analysis of 12 nominated lncRNAs. Three NETs-related lncRNAs (AP000695.2, MYOSLID, NKILA) were as independent adverse factors for NSCLC. The other lncRNAs, that represented independent favorable factors, were as follows: AC020765.2, AC090152.1, AF131215.5, AL035587.1, AP004608.1, MMP2.AS1, SCAMP1.AS1, SNHG10, and TRG.AS.

A total of 119 NSCLC and NETs-related genes were identified, as summarized above, and 13413 lncRNAs were referred to in TCGA. A NETs-related gene lncRNA co-expression network was constructed to identify the NETs-related lncRNAs, and 1039 NETs-related lncRNAs were selected (|*R*^2^| ≥ 0.3 and *P* ≤ 0.001). Subsequently, according to the univariate Cox regression analysis, 19 NETs-related lncRNAs had prognostic value for patients with lung cancer (*P* < 0.05, Figure 3B, 3C). As a result of Lasso Cox regression, 12 NETs-related lncRNAs were identified (Supplementary Figure 1), among which three lncRNAs (AP000695.2, MYOSLID, and NKILA) were considered as harmful prognostic factors, while the others (AC020765.2, AC090152.1, AF131215.5, AL035587.1, AP004608.1, MMP2.AS1, SCAMP1.AS1, SNHG10, and TRG.AS) were considered as favorable prognostic factors (Figure 4A–4L). Ultimately, a NETs-related co-expression network consisting of 12 lncRNAs revealed the correlations between the genes and lncRNAs referenced above (Figure 3D, 3E).

### Establishment of a prognostic model in the training cohort

**Figure 5:**
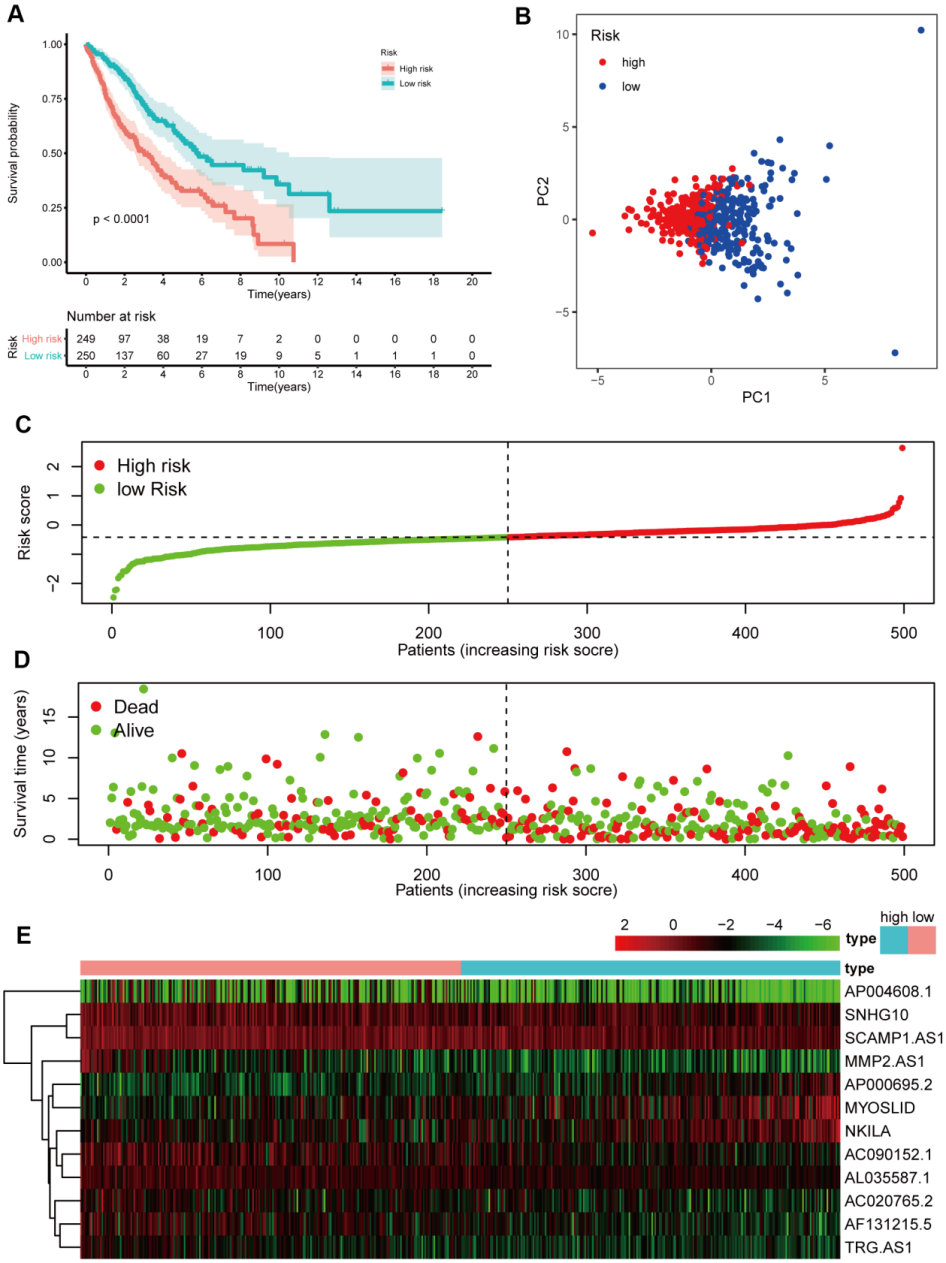
**Prognostic analyses of the 12 NETs-related lncRNA signature model in the training cohort.** (**A**) Kaplan–Meier survival analysis based on 12 NETs-related lncRNAs in the high-risk group and low-risk groups. (**B**) Principal component analysis (PCA) plot of the training cohort. (**C**) The distribution and median values of the risk scores in different groups. (**D**) The distributions of survival time in the training cohort. (**E**) Heatmap of the expression levels of the 12 lncRNAs related to OS.

A prognostic model based on the 19 lncRNAs referenced above was built following univariate Cox regression analysis, and 12 lncRNAs were determined through Lasso Cox regression based on 19 lncRNAs. The formula, risk score = 0.044 * expression level of AC020765.2 + 0.079 * expression level of AC090152.1 + 0.133 * expression level of AF131215.5 + 0.067 * expression level of AL035587.1 + 0.044 * expression level of AP000695.2 + 0.059 * expression level of AP004608.1 + 0.216 * expression level of MMP2.AS1 + 0.006 * expression level of MYOSLID + 0.076 * expression level of NKILA + 0.018 * expression level of SCAMP1.AS1+ 0.027 * expression level of SNHG10+ 0.063 * expression level of TRG.AS, was utilized to calculate the risk score. By evaluating these 12 NETs-related lncRNAs, we were able to acquire the risk score of each patient. The patients were divided into a high-risk group (n=241) and a low-risk group (n=241) in accordance with the risk score (Figure 5C). Next, the Kaplan-Meier curve suggested that patients in the high-risk group had a significantly worse OS than their low-risk counterparts (Figure 5A, *P* < 0.05). Additionally, PCA indicated that the patients in different risk groups were distributed in two directions (Figure 5B). Patients with high risk had a higher probability of earlier death than those with low risk (Figure 5D, 5E).

### Verification of the 12 NETs-related lncRNA signature in the validation cohort

**Figure 6:**
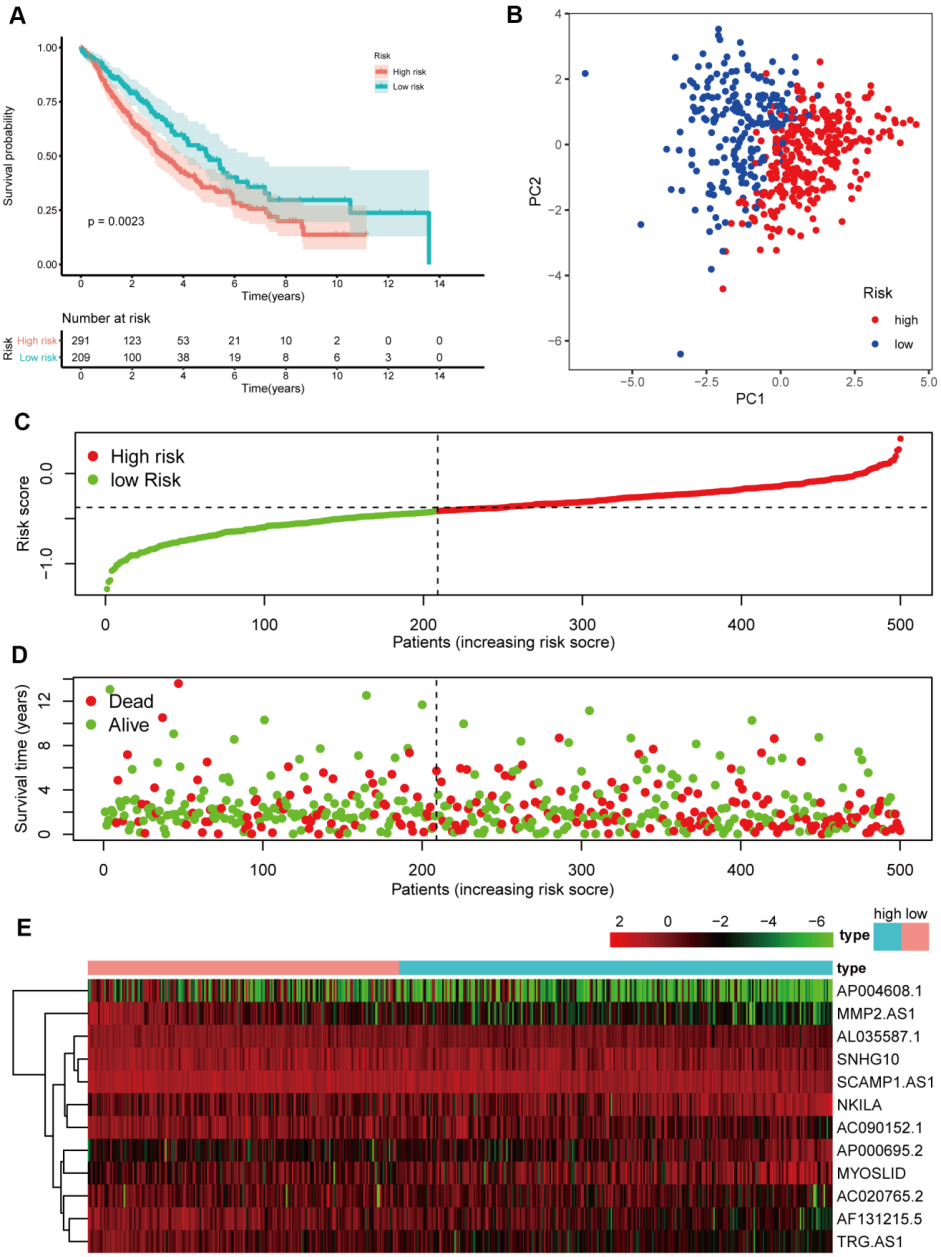
**Prognostic analyses of the validation cohort.** (**A**) Kaplan–Meier survival analysis. (**B**) PCA plot. (**C**) The risk scores in the different groups. (**D**) The survival time in the training cohort. (**E**) Heatmap of the expression levels of the 12 lncRNAs related to OS.

To test the strength of the model built from the training cohort, the patients in the validation cohort were also categorized into high- and low-risk groups by the median value calculated with the same formula as that of the training cohort. Similar to the results obtained from the training cohort, patients in the high-risk group, had a reduced survival time, compared with those in the low-risk group (Figure 6A, *P* < 0.05) and were more likely to die earlier (Figure 6D, 6E). Additionally, PCA confirmed that patients in the two subgroups were distributed in discrete directions (Figure 6B).

### Independent prognostic value of the 12 NETs-related lncRNA signature in both two cohorts

**Figure 7:**
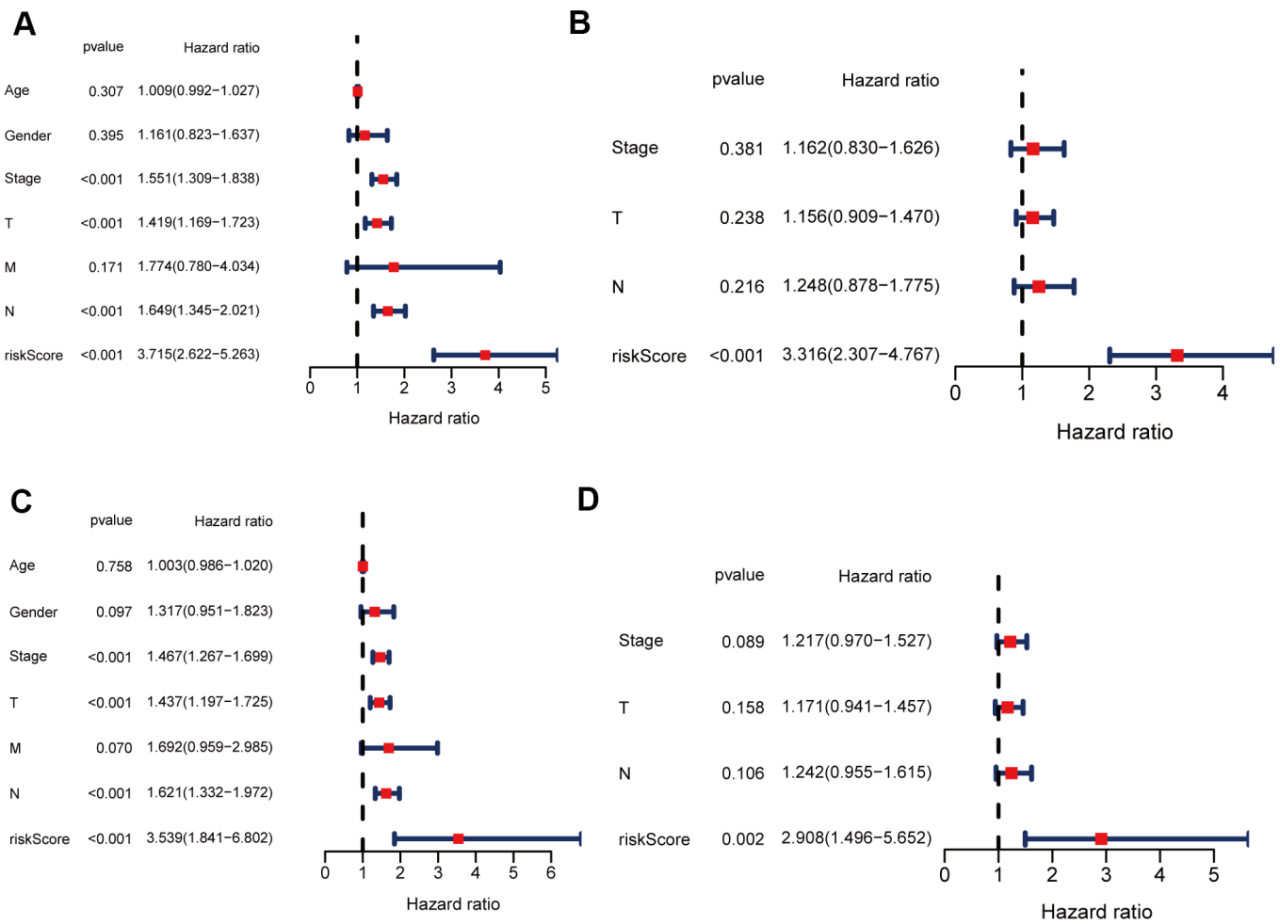
**Univariate and multivariate Cox regression analyses for OS.** (**A**, **B**) Analyses of the training cohort. (**C**, **D**) Analyses of validation cohort.

Univariate and multivariate Cox regression analyses were carried out among the available variables to determine whether the risk score was an independent prognostic predictor for OS. In univariate Cox regression analyses, the risk score was significantly associated with OS in both the training and validation cohorts (HR = 3.715, 95% CI = 2.622-5.263, *P* < 0.05; HR = 3.539, 95% CI = 1.841-6.802, *P* < 0.05, respectively) (Figure 7A, 7C). After correction for other confounding factors, the risk score still proved to be an independent predictor for OS in the multivariate Cox regression analysis (training cohort: HR = 3.316, 95% CI = 2.307-4.767, *P* < 0.05; test cohort: HR = 2.908, 95% CI = 1.496-5.652, *P* < 0.05; Figure 7B, 7D).

### Validation of the expression of the 3 adverse prognostic lncRNAs at the NSCLC cell level

**Figure 8:**
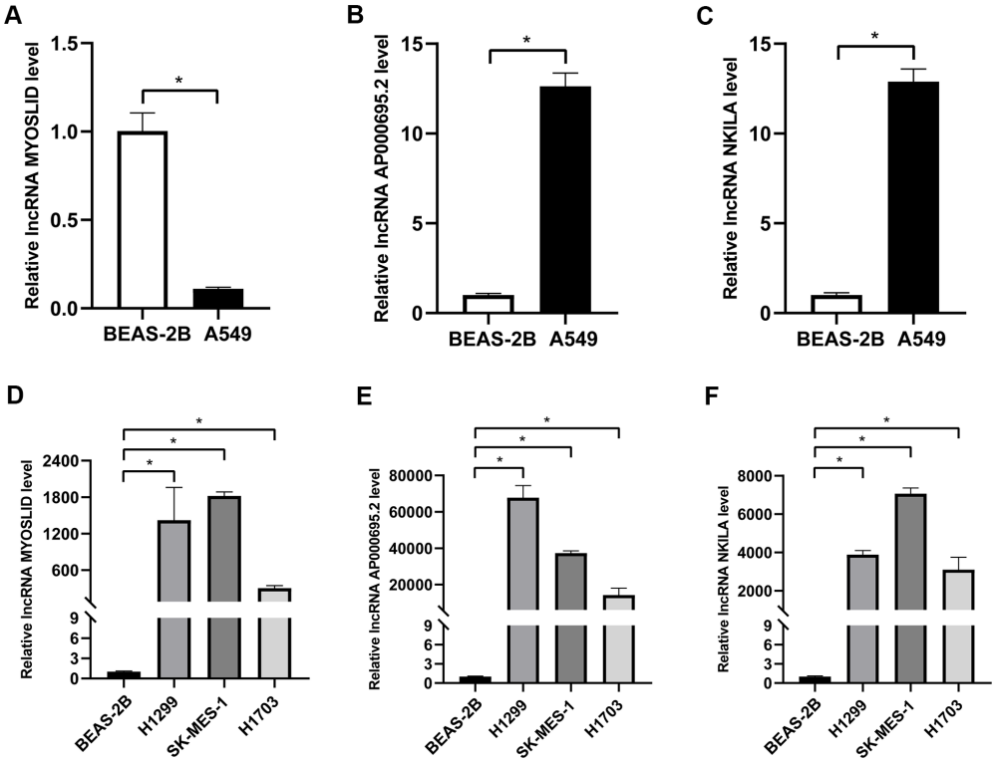
**Validation of the expression levels of 3 adverse prognostic lncRNAs in different NSCLC cells by RT-qPCR.** (**A**–**F**) Expression levels of lncRNA AP000695.2, MYOSLID, and NKILA in human normal lung epithelial cells (BEAS-2B) and NSCLC cells (A549, H1299, SK-MES-1, and H1703), and presented as bar charts. ^*^P < 0.05.

**Figure 9:**
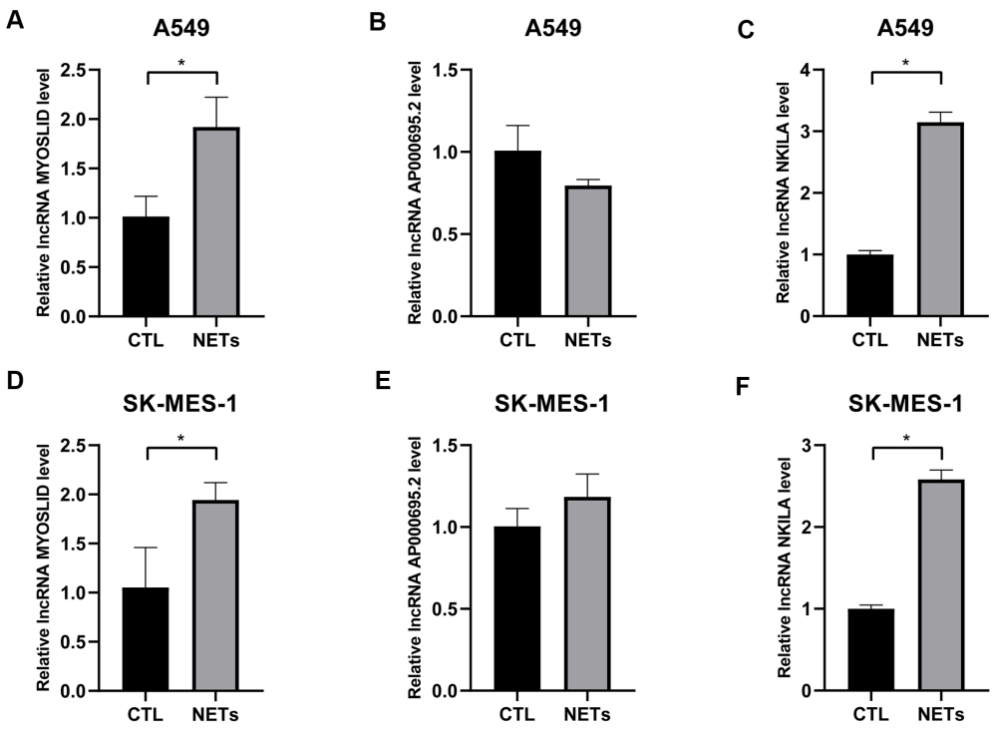
**Validation of the expression levels of 3 adverse prognostic lncRNAs treated with NETs by RT-qPCR.** (**A**–**F**) Expression levels of lncRNA AP000695.2, MYOSLID, and NKILA in NSCLC cells (A549, and SK-MES-1) treated with or without NETs for 12h, and presented as bar charts. ^*^P < 0.05.

As mentioned before, survival data analyses clearly revealed that AP000695.2, MYOSLID, and NKILA were unfavorable prognostic factors. Here, we conducted further verification to understand the characteristics of these 3 lncRNAs at the cell level. As shown in Figure 8A–8C, the levels of AP000695.2 and NKILA in A549 cell were much higher than those in human normal epithelial lung cells (BEAS-2B), whereas the expression of MYOSLID in the A549 cells was lower. The expression levels of 3 lncRNAs, namely, AP000695.2, MYOSLID, and NKILA, in NSCLC cells (H1299, SK-MES-1, H1703) was distinctly higher than those in BEAS-2B, as detected by RT-qPCR (Figure 8D–8F). Therefore, we tested the 3 lncRNAs that were expressed at higher levers in A549 and SK-MES-1 cells following NETs treatment or not for 12 h (Figure 9A–9F), and the results showed that the expression levels of MYOSLID and NKILA were elevated in both NSCLC cells lines after NETs treatment for 12 h (*P* < 0.05).

## DISCUSSION

Non-small cell lung cancer, a primary thoracic malignancy worldwide, is the leading cause of cancerous death, thus, it is urgent to find reliable molecular biomarkers that can predict the prognosis of NSCLC to improve the survival rate [2, 17]. NETs have been reported to play complicated and momentous roles in NSCLC progression [18]. NETs can regulate cell activities and metabolism by inducing tumor microenvironment heterogeneity and chiefly promote tumor progression by setting the premetastatic niche, such as capturing circulating tumor cells (CTCs) and inducing epithelial-mesenchymal transition (EMT) [19]. LncRNAs, as a significant kind of noncoding RNAs, have an intimate connection with the genomes that impacts the transformed phenotype of cancer cells in terms of cell cycle variation, survival, immune response, and other processes [20]. Moreover, because the expression levels of lncRNAs were found to be different in tumors, they became one of the direct reasons for the normal cells to convert into tumor cells [21, 22], and play vital functions in cancer diagnosis and prognosis as new biomarkers [14, 23]. However, the prognostic significance of related lncRNAs in NSCLC accepting NETs has not been covered. Here, we established a 12 NETs-related lncRNA signature model to predict the prognosis of NSCLC patients.

In the present research, we comprehensively examined NETs-related lncRNAs by building a co-expression network between lncRNAs and NETs-related genes. Furthermore, 12 prognostic NETs-related lncRNAs were identified through Lasso Cox regression as follow: AC020765.2, AC090152.1, AF131215.5, AL035587.1, AP000695.2, AP004608.1, MMP2.AS1, MYOSLID, NKILA, SCAMP1.AS1, SNHG10, and TRG.AS. A fresh prognostic model was constructed based on these lncRNAs and was tested in a validation cohort, and all showed the potential of being prognostic biomarkers. Six NETs-related lncRNAs (AF131215.5, MYOSLID, NKILA, AC090152.1, SNHG10, and TRG.AS) have been reported to be associated with cancer progression. AF131215.5 was found to have an independent prognostic value of overall survival for patients with lung adenocarcinoma (LUAD) [24]. In terms of partial epithelial-mesenchymal transition (p-EMT), MYOSLID expression in head and neck squamous cell carcinoma (HNSCC) was closely correlated with Slug, PDPN, and LAMB3, and is a key regulator of tumor cell survival. Knockout of MYOSLID in the HNSCC cell lines Cal27, SCC4 and SCC9 significantly inhibited migration and invasion [25]. NKILA is considered part of a class of NF-κB modulators that suppress cancer metastasis [26], whereas Huang et al [27] reported that NKILA could sensitize T cells to activation-induced cell death (AICD) which can promote tumor immune evasion. Research [28] has shown that AC090152.1 is capable of effectively predicting the overall survival (OS) in HCC patients with or without fibrosis. As novel drivers of the malignant phenotype of HCC, SNHG10 [29] and its homolog SCARNA13, which form a positive feedback loop, coordinately contribute to the cancer development. High expression of TRG.AS [30], results in poorer survival than in patients with low expression, as TRG.AS serves as a molecular sponge for microRNA-543 (miR-543), thereby mechanistically contributing to the increased expression of Yes-associated protein 1 (YAP1). For the six remaining NETs-related lncRNAs (AC020765.2, SCAMP1.AS1, AL035587.1, AP000695.2, AP004608.1, and MMP2.AS1), there have been no studies exploring their potential roles in the development of cancer at present. Regrettably, there are still few reports revealing the association between the 12 lncRNAs mentioned above and NSCLC, reports on how the mutual effect of lncRNAs with NETs-related genes are even unusual. Therefore, it is essential to carry out correlational research in the future. For this reason, more research is necessary to explore whether these lncRNAs are closely connected with the prognosis of NSCLC patients after NETs are stimulated.

A signature-based on 12 NETs-related lncRNAs significantly predicted the prognosis of NSCLC patients. In keeping with former studies [31, 32], the OS of the low-risk group was longer than that of the high-risk group. This phenomenon reminded us that the risk score signature had a definite ability to forecast survival. Remarkably, we observed identical results in the validation cohort. The independent prognostic value of the signature was corroborated by employing both univariate and multivariate Cox analyses.

Our study observed that the expression levels of 3 adverse prognostic lncRNAs (AP000695.2, MYOSLID, and NKILA) in NSCLC cells tended to be higher than in normal lung epithelial cells, which is in accordance with previous research [28, 33]. Moreover, the expression levels of the lncRNAs rose distinctly with NETs treatment, compared to the untreated group, which verified the prognostic model outlined above. Hence, it is our hope that our discoveries will contribute to identifying the prognostic lncRNAs related to NETs stimulation, offering opinions on their possible roles in NSCLC tumorigenesis and progression.

There are several limitations in this study. First of all, without functional enrichment analyses, the potential molecular mechanisms of NETs-related lncRNAs in prediction remains unclear. Second, more prospective studies are needed to confirm the prognostic function of NETs-related lncRNAs because our research is a retrospective study. Third, we only tested our conclusion at the cell level. Animal experiments and even human studies are warranted to verify its clinical utility. In addition, we tested three adverse prognostic lncRNAs treated with NETs by RT-qPCR. Focusing on validated target lncRNAs may exclude potential targets that the experiment has not validated. In brief, we conducted all-sided bioinformatics analysis of NETs-related lncRNAs in NSCLC. Our research detected 12 NETs-related lncRNAs deemed to be distinctly associated with the prognosis of the NSCLC patients. A NETs-related lncRNA model consisting of the lncRNAs above was regarded as independently associated with OS in both the training and validation cohorts, offering insight into the forecast of NSCLC prognosis proven in cell experiments. Hence, the model outlined suggests that the 12 NETs-related lncRNAs identified could play an influential role as molecular markers in NSCLC.

## MATERIALS AND METHODS

### Cell culture

The human bronchial epithelial cells line (BEAS-2B), and human lung adenocarcinoma cells (A549 and H1299), human squamous cell carcinoma cell lines (SK-MES-1 and H1703), purchased from the Type Culture Collection of the Chinese Academy of Sciences, Shanghai, China, were all grown in high glucose DMEM medium (BI, Israel) supplemented with 10% fetal bovine serum (Gibco, Grand Island, USA), penicillin (100 U/ml), and streptomycin (100 μg/ml). The cells were incubated in a humidified atmosphere containing 5% CO^2^ at 37° C.

### NETs isolation and treatment

NETs were obtained from rat neutrophils following phorbol 12-myristate-13-acetate (PMA) 100 uM stimulated 4h as previous studies [34, 35]. A549 cells were randomly divided into two groups (each group n=3): without NETs-stimulated group, NETs-stimulated 12h group. TRIzol reagent (Invitrogen, Carlsbad, USA) was used to isolate and purify the total RNA of A549 cells. Then, RNA samples, A549 cells with or without NETs treated 12h, were sent to the laboratory of the OE Biotech Company (Shanghai, China) and the microarray profiling was carried out.

### Data and sample collection

The RNA sequencing (RNA-seq) data of 1037 NSCLC patients and 108 healthy people samples were received from the TCGA database (https://portal.gdc.cancer.gov/repository). Additionally, the detailed clinical materials of partial patients were obtained from TCGA. Patients without clinical data recorded were eliminated when the related clinical prognostic analysis was performed. Both NSCLC patients and healthy people samples fell into two groups at random equally and served as training and validation data sets, respectively. The gene expression profiles data from TCGA were normalized, and normalization was not needed. The data from TCGA is openly getable, so that the present study was not required the permission of local ethics committees. The current study abides by the access policies and publication guidelines of TCGA.

The NETs-related genes were obtained from the transcriptome RNA microarray data of A549 cells with or without NETs treated 12h.

### Identification of NETs-related genes and lncRNAs

"limma" R package was exploited to identify the differentially expressed genes (DEGs) between NSCLC and healthy people with | log2(Fold Change) | ≥ 1, and FDR < 0.05, in the training cohort, and the differentially expressed NETs-related genes from the transcriptome RNA microarray were screened in the same way. Then, prognostic NETs-related genes were selected by using Venny 2.1.0 based on further analysis of genes aforesaid. Subsequently, Pearson correlation was utilized to measure the relationship between lncRNAs and NETs-related genes. NETs-related lncRNAs were ensured according to the square of correlation coefficient |*R*^2^| > 0.3 and *P* < 0.05.

### Establishment and test of a prognostic NETs-related lncRNA signature

NETs-related lncRNAs with prognostic values were picked out via univariate Cox analysis of overall survival (OS). *P* values were adjusted by Benjamini and Hochberg (BH) correction. The Lasso Cox regression analysis [33, 36] constructed an NSCLC prognostic model to lessen the chance of overfitting as much as possible. The Lasso algorithm and "glmnet" R package were utilized for selecting variable and shrinkage together. The normalized expression array of prognostic NETs-related lncRNAs was the independent variable in the regression, and the dependent variables were the overall survival and status of patients in the training cohort. Then, the Co-expression networks were depicted through Cytoscape software 3.7.2.

Judging by the normalized expression level of each lncRNA and its homologous regression coefficients, the risk scores of the NSCLC patients were gained. The formula was operated: score = sum (each gene’s expression × corresponding coefficient). The patients were divided into high- and low-risk groups severally according to the median value of the risk score. PCA was performed with the "ggplot2" function in the "Rtsne " R package in light of the expression of lncRNAs in the signature. The ideal cut-off expression value was calculated via the "surv_cutpoint" function of the "survminer" R package to achieve survival analysis for each lncRNA.

### Real-time quantitative polymerase chain reaction

Total RNA from the BEAS-2B, A549, H1299, SK-MES-1, and H1703 cell, according to the manufacturer’s specification, was separated using Trizol (Invitrogen, Carlsbad, USA). Then, the first-strand cDNA at a volume of 20μL was compound by using the reverse transcription kit and finally diluted to 100uL. Quantitative real-time polymerase chain reaction (RT-qPCR) analyses were processed utilizing an SYBR Green mix in the Real-Time PCR System (TAKARA, Kyoto, Japan). 20μL of PCR reaction was prepared as follows: 10μL of 2×SYBR Green PCR master mix, 1 μL of 10μM of suitable forward and reverse primers, 7μL of RNase-free water, and 2μL of cDNA template. RT-qPCR was performed for 10 minutes at 95° C, followed by 40 cycles of 5 seconds at 95° C and 30 seconds at 60° C. A dissociation melting curve was generated using thermal conditions from 60° C to 95° C. Human GAPDH was employed as an internal housekeeping reference. The sequences for all primers were as follows: NKILA (forward primer): GGCTAGTCTGGCTGGGAGAAGTC; (reverse primer): AGCGTTGTGGGTAGGTTTGGTTTC. MYOSLID (forward primer): TCTGCCTAGTCCTGCTGCCTTC; (reverse primer): ATGGGAAGCTGTGTTCACTTTGGG; AP000695.2 (forward primer): CGGAAGCCACCACATGACCTTG; (reverse primer): TTCCAACCGCATGGGTGAAAGTC. GAPDH (forward primer): GTCAGTGGTGGACTGACCT; (reverse primer): TGCTGTAGCCAAATTCGTTG.

### Statistical analysis

The OS between different groups was compared by Kaplan-Meier analysis with the log-rank test. Univariate and multivariate Cox regression analyses were implemented to identify independent predictors of OS. All statistical analyses were finished with R software (Version 3.5.3) or GraphPad Prism 8 (Version 8.0). If not particular remark, *P* < 0.05 was considered statistically significant, and all *P* values were two-tailed.

## Supplementary Material

Supplementary Figure 1

## References

[1] Siegel RL, Miller KD, Jemal A. Cancer statistics, 2020. CA Cancer J Clin. 2020; 70:7–30. 10.3322/caac.21590 31912902

[2] Reck M, Rabe KF. Precision Diagnosis and Treatment for Advanced Non-Small-Cell Lung Cancer. N Engl J Med. 2017; 377:849–61. 10.1056/NEJMra1703413 28854088

[3] Rizvi NA, Hellmann MD, Snyder A, Kvistborg P, Makarov V, Havel JJ, Lee W, Yuan J, Wong P, Ho TS, Miller ML, Rekhtman N, Moreira AL, et al. Cancer immunology. Mutational landscape determines sensitivity to PD-1 blockade in non-small cell lung cancer. Science. 2015; 348:124–28. 10.1126/science.aaa1348 25765070 PMC4993154

[4] Herbst RS, Morgensztern D, Boshoff C. The biology and management of non-small cell lung cancer. Nature. 2018; 553:446–54. 10.1038/nature25183 29364287

[5] Ravindran M, Khan MA, Palaniyar N. Neutrophil Extracellular Trap Formation: Physiology, Pathology, and Pharmacology. Biomolecules. 2019; 9:365. 10.3390/biom9080365 31416173 PMC6722781

[6] Cedervall J, Zhang Y, Olsson AK. Tumor-Induced NETosis as a Risk Factor for Metastasis and Organ Failure. Cancer Res. 2016; 76:4311–15. 10.1158/0008-5472.CAN-15-3051 27402078

[7] Jorch SK, Kubes P. An emerging role for neutrophil extracellular traps in noninfectious disease. Nat Med. 2017; 23:279–87. 10.1038/nm.4294 28267716

[8] Papayannopoulos V. Neutrophil extracellular traps in immunity and disease. Nat Rev Immunol. 2018; 18:134–47. 10.1038/nri.2017.105 28990587

[9] Wang W, Zhang J, Zheng N, Li L, Wang X, Zeng Y. The role of neutrophil extracellular traps in cancer metastasis. Clin Transl Med. 2020; 10:e126. 10.1002/ctm2.126 32961033 PMC7580875

[10] Yang L, Liu Q, Zhang X, Liu X, Zhou B, Chen J, Huang D, Li J, Li H, Chen F, Liu J, Xing Y, Chen X, et al. DNA of neutrophil extracellular traps promotes cancer metastasis via CCDC25. Nature. 2020; 583:133–38. 10.1038/s41586-020-2394-6 32528174

[11] Kopp F, Mendell JT. Functional Classification and Experimental Dissection of Long Noncoding RNAs. Cell. 2018; 172:393–407. 10.1016/j.cell.2018.01.011 29373828 PMC5978744

[12] Huarte M. The emerging role of lncRNAs in cancer. Nat Med. 2015; 21:1253–61. 10.1038/nm.3981 26540387

[13] Lu W, Zhang H, Niu Y, Wu Y, Sun W, Li H, Kong J, Ding K, Shen HM, Wu H, Xia D, Wu Y. Long non-coding RNA linc00673 regulated non-small cell lung cancer proliferation, migration, invasion and epithelial mesenchymal transition by sponging miR-150-5p. Mol Cancer. 2017; 16:118. 10.1186/s12943-017-0685-9 28697764 PMC5504775

[14] Acha-Sagredo A, Uko B, Pantazi P, Bediaga NG, Moschandrea C, Rainbow L, Marcus MW, Davies MP, Field JK, Liloglou T. Long non-coding RNA dysregulation is a frequent event in non-small cell lung carcinoma pathogenesis. Br J Cancer. 2020; 122:1050–58. 10.1038/s41416-020-0742-9 32020063 PMC7109049

[15] Zhou Y, Shi H, Du Y, Zhao G, Wang X, Li Q, Liu J, Ye L, Shen Z, Guo Y, Huang Y. lncRNA DLEU2 modulates cell proliferation and invasion of non-small cell lung cancer by regulating miR-30c-5p/SOX9 axis. Aging (Albany NY). 2019; 11:7386–401. 10.18632/aging.102226 31541993 PMC6781974

[16] Zhang W, Cai X, Yu J, Lu X, Qian Q, Qian W. Exosome-mediated transfer of lncRNA RP11-838N2.4 promotes erlotinib resistance in non-small cell lung cancer. Int J Oncol. 2018; 53:527–38. 10.3892/ijo.2018.4412 29845246 PMC6017264

[17] Bray F, Ferlay J, Soerjomataram I, Siegel RL, Torre LA, Jemal A. Global cancer statistics 2018: GLOBOCAN estimates of incidence and mortality worldwide for 36 cancers in 185 countries. CA Cancer J Clin. 2018; 68:394–24. 10.3322/caac.21492 30207593

[18] Cools-Lartigue J, Spicer J, McDonald B, Gowing S, Chow S, Giannias B, Bourdeau F, Kubes P, Ferri L. Neutrophil extracellular traps sequester circulating tumor cells and promote metastasis. J Clin Invest. 2013; 123:3446–58. 10.1172/JCI67484 23863628 PMC3726160

[19] Brostjan C, Oehler R. The role of neutrophil death in chronic inflammation and cancer. Cell Death Discov. 2020; 6:26. 10.1038/s41420-020-0255-6 32351713 PMC7176663

[20] Yao RW, Wang Y, Chen LL. Cellular functions of long noncoding RNAs. Nat Cell Biol. 2019; 21:542–51. 10.1038/s41556-019-0311-8 31048766

[21] Lv X, Lian Y, Liu Z, Xiao J, Zhang D, Yin X. Exosomal long non-coding RNA LINC00662 promotes non-small cell lung cancer progression by miR-320d/E2F1 axis. Aging (Albany NY). 2021; 13:6010–24. 10.18632/aging.202522 33589572 PMC7950287

[22] Sun CC, Zhu W, Li SJ, Hu W, Zhang J, Zhuo Y, Zhang H, Wang J, Zhang Y, Huang SX, He QQ, Li DJ. FOXC1-mediated LINC00301 facilitates tumor progression and triggers an immune-suppressing microenvironment in non-small cell lung cancer by regulating the HIF1α pathway. Genome Med. 2020; 12:77. 10.1186/s13073-020-00773-y 32878637 PMC7466809

[23] Ye R, Tang R, Gan S, Li R, Cheng Y, Guo L, Zeng C, Sun Y. New insights into long non-coding RNAs in non-small cell lung cancer. Biomed Pharmacother. 2020; 131:110775. 10.1016/j.biopha.2020.110775 33152934

[24] Hou J, Yao C. Potential Prognostic Biomarkers of Lung Adenocarcinoma Based on Bioinformatic Analysis. Biomed Res Int. 2021; 2021:8859996. 10.1155/2021/8859996 33511215 PMC7822677

[25] Xiong HG, Li H, Xiao Y, Yang QC, Yang LL, Chen L, Bu LL, Zhang WF, Zhang JL, Sun ZJ. Long noncoding RNA MYOSLID promotes invasion and metastasis by modulating the partial epithelial-mesenchymal transition program in head and neck squamous cell carcinoma. J Exp Clin Cancer Res. 2019; 38:278. 10.1186/s13046-019-1254-4 31238980 PMC6593600

[26] Liu B, Sun L, Liu Q, Gong C, Yao Y, Lv X, Lin L, Yao H, Su F, Li D, Zeng M, Song E. A cytoplasmic NF-κB interacting long noncoding RNA blocks IκB phosphorylation and suppresses breast cancer metastasis. Cancer Cell. 2015; 27:370–81. 10.1016/j.ccell.2015.02.004 25759022

[27] Huang D, Chen J, Yang L, Ouyang Q, Li J, Lao L, Zhao J, Liu J, Lu Y, Xing Y, Chen F, Su F, Yao H, et al. NKILA lncRNA promotes tumor immune evasion by sensitizing T cells to activation-induced cell death. Nat Immunol. 2018; 19:1112–25. 10.1038/s41590-018-0207-y 30224822

[28] Ye J, Wu S, Pan S, Huang J, Ge L. Risk scoring based on expression of long non-coding RNAs can effectively predict survival in hepatocellular carcinoma patients with or without fibrosis. Oncol Rep. 2020; 43:1451–66. 10.3892/or.2020.7528 32323856 PMC7108035

[29] Lan T, Yuan K, Yan X, Xu L, Liao H, Hao X, Wang J, Liu H, Chen X, Xie K, Li J, Liao M, Huang J, et al. LncRNA SNHG10 Facilitates Hepatocarcinogenesis and Metastasis by Modulating Its Homolog SCARNA13 via a Positive Feedback Loop. Cancer Res. 2019; 79:3220–34. 10.1158/0008-5472.CAN-18-4044 31101763

[30] He S, Wang X, Zhang J, Zhou F, Li L, Han X. TRG-AS1 is a potent driver of oncogenicity of tongue squamous cell carcinoma through microRNA-543/Yes-associated protein 1 axis regulation. Cell Cycle. 2020; 19:1969–82. 10.1080/15384101.2020.1786622 32615889 PMC7469544

[31] Li JP, Li R, Liu X, Huo C, Liu TT, Yao J, Qu YQ. A Seven Immune-Related lncRNAs Model to Increase the Predicted Value of Lung Adenocarcinoma. Front Oncol. 2020; 10:560779. 10.3389/fonc.2020.560779 33163400 PMC7591457

[32] Zhu Q, Yang H, Cheng P, Han Q. Bioinformatic analysis of the prognostic value of the lncRNAs encoding snoRNAs in hepatocellular carcinoma. Biofactors. 2019; 45:244–52. 10.1002/biof.1478 30537372

[33] Simon N, Friedman J, Hastie T, Tibshirani R. Regularization Paths for Cox’s Proportional Hazards Model via Coordinate Descent. J Stat Softw. 2011; 39:1–13. 10.18637/jss.v039.i05 27065756 PMC4824408

[34] Najmeh S, Cools-Lartigue J, Giannias B, Spicer J, Ferri LE. Simplified Human Neutrophil Extracellular Traps (NETs) Isolation and Handling. J Vis Exp. 2015; 98:52687. 10.3791/52687 25938591 PMC4541576

[35] Fuchs TA, Brill A, Duerschmied D, Schatzberg D, Monestier M, Myers DD Jr, Wrobleski SK, Wakefield TW, Hartwig JH, Wagner DD. Extracellular DNA traps promote thrombosis. Proc Natl Acad Sci USA. 2010; 107:15880–85. 10.1073/pnas.1005743107 20798043 PMC2936604

[36] Tibshirani R. The lasso method for variable selection in the Cox model. Stat Med. 1997; 16:385–95. 10.1002/(sici)1097-0258(19970228)16:4<385::aid-sim380>3.0.co;2-3 9044528

