# Supplementary material for: A innovative prognostic symbol based on neutrophil extracellular traps (NETs)-related lncRNA signature in non-small-cell lung cancer
Source: Aging (Albany NY). 2021 Jul 13;13(13):17864–79. doi: 10.18632/aging.203289 (PMC8312458; doi:10.18632/aging.203289)
Supplement: Supplementary Figure 1 [file aging-13-203289-s001.pdf]

SUPPLEMENTARY FIGURE

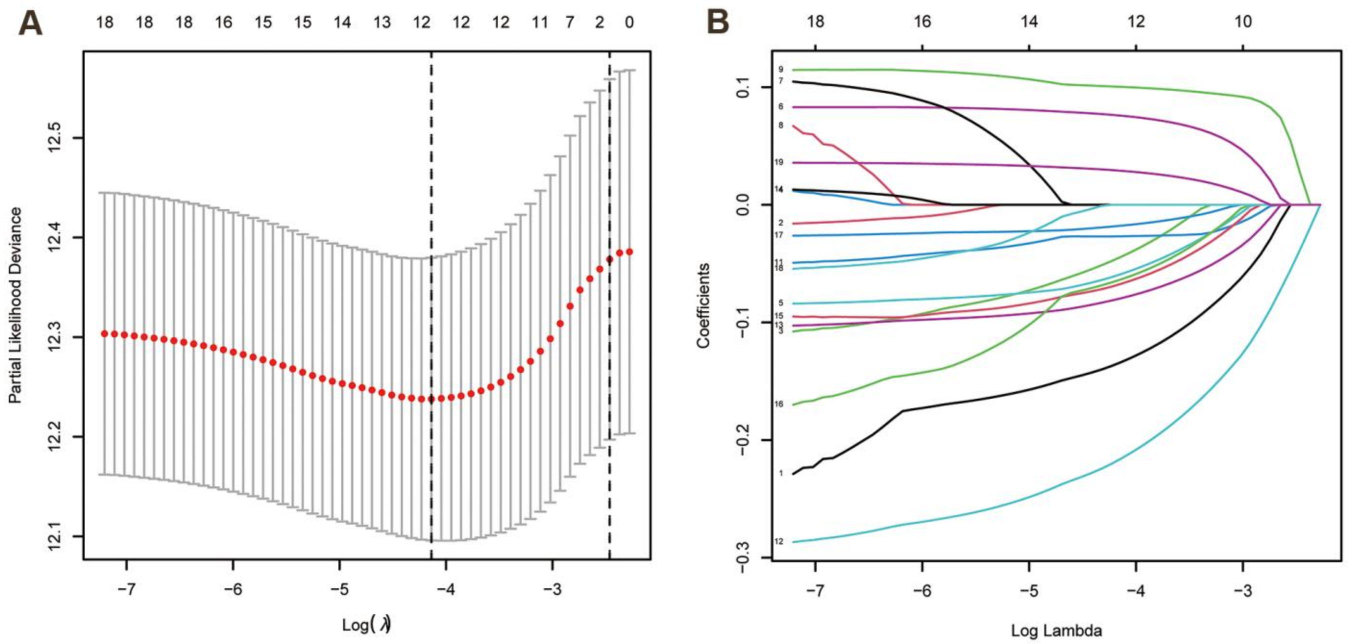

Supplementary Figure 1. Construction of NETs-related lncRNA signature model by Lasso model. (**A**) Lasso coefficient values of the expression of 19 NETs-related lncRNAs. Optimal penalty parameter log (λ) value were chosen in the Lasso model. (**B**) Lasso coefficient profiles of 19 NETs-related lncRNAs.
